# Sequencing of DC-SIGN promoter indicates an association between promoter variation and risk of nasopharyngeal carcinoma in cantonese

**DOI:** 10.1186/1471-2350-11-161

**Published:** 2010-11-11

**Authors:** Ya-Fei Xu, Wan-Li Liu, Ju-Qin Dong, Wen-Sheng Liu, Qi-Sheng Feng, Li-Zhen Chen, Yi-Xin Zeng, Mu-Sheng Zeng, Wei-Hua Jia

**Affiliations:** 1 State Key Laboratory of Oncology in South China, 651 Dongfeng Road East, Guangzhou 510060, China; 2 Department of Experimental Research, Sun Yat-sen University Cancer Center, 651 Dongfeng Road East, Guangzhou 510060, China

## Abstract

**Background:**

The dendritic cell-specific intercellular adhesion molecule 3 grabbing non-integrin (*DC-SIGN*) is an important pathogen recognition receptor of the innate immune system. *DC-SIGN *promoter variants play important role in the susceptibility to various infectious diseases. Nasopharyngeal carcinoma (NPC) is a malignancy that is common in southern China and whether *DC-SIGN *promoter variants have effects on susceptibility to NPC is still unknown. The aim of this study is to ascertain the potential involvement of *DC-SIGN *promoter single nucleotide polymorphisms (SNPs) in NPC susceptibility.

**Methods:**

We conducted a case control study based on Cantonese population including 444 NPC patients and 464 controls matched on age and sex. The 1041 bp of *DC-SIGN *promoter region was directly sequenced for all samples. Sequence alignment and SNP search were inspected using DNAStar analysis programs and haplotype frequencies were estimated in Haploview V 4.0. The associations between the SNPs and the risk of NPC were analyzed using chi-square test and non-conditional logistic regression analysis with SPSS 13.0 software.

**Results:**

A total of six variants were observed in the *DC-SIGN *promoter region and *DC-SIGN *-139 GG and -939 AA were significantly associated with NPC risk with adjusted Odds Ratios (ORs) of 2.10 (95% confidence interval [CI] = 1.23-3.59; *P *= 0.006) and 2.52 (1.29-4.93; *P *= 0.007) respectively and subjects carrying the risk allele *DC-SIGN *-871 G had 1.47-fold (95% CI = 1.14-1.90) increased risks of developing NPC (*P *= 0.003). Haplotype analysis revealed that h1 'AAAG' was significantly associated with protection against NPC (OR = 0.69; *P *= 0.0002) and the association was still significant when using 1000 permutation test runs (*P *= 0.001).

**Conclusions:**

Our study indicated that *DC-SIGN *promoter variants appear to be involved in the susceptibility to NPC and the detailed mechanism of this effect need further studies.

## Background

Nasopharyngeal carcinoma (NPC) is rare in most populations around the world but common in China and Southeast Asia, where the incidence can reach 20 to 50 per 100,000 individuals [1-5]. Epstein-Barr virus (EBV) is considered a major risk factor for NPC, and there is a dose-response relationship between EBV antibodies and NPC risk [6,7]. EBV is present in over 90% of the world population [8], most often as a form of in vivo latency in healthy carriers with low copies of episomal virus maintained in resting memory B cells [9-11].

*DC-SIGN *(Dendritic cell-specific intercellular adhesion molecule 3 grabbing non-integrin), encoded by CD209 on chromosome 19p13.3, is a C-type lectin that is expressed on subsets of dendritic cells (DCs) and alveolar macrophages [12-15], and functions both as a cell adhesion receptor and as a pathogen recognition receptor [16]. Acting as a pathogen uptake receptor, *DC-SIGN *could mediate interactions with a plethora of pathogens [17] including bacteria such as Helicobacter pylori [18]; viruses such as HIV-1 [19], Ebola [20,21], Cytomegalovirus [22], Hepatitis-C virus [23,24], Dengue virus [20,21,25], and SARS-coV [26], and parasites such as Leishmania pifanoi [27]. Several studies have recently reported on the role of *DC-SIGN *promoter variants in the susceptibility to or pathogenesis of various infectious diseases, such as dengue fever [25,28], tuberculosis [13,14,29,30], Acquired Immune Deficiency Syndrome (AIDS) [19,31-33], celiac disease [34]. Sakuntabhai et al. [25] reported that the G allele of the variant *DC-SIGN *-336 was associated with strong protection against dengue fever [35]. In addition, several previous reports suggested that variants in the *DC-SIGN *promoter conferred protection against tuberculosis [29,30]. However, whether *DC-SIGN *promoter variants have effects on susceptibility to NPC is still unknown and so far no study has reported on the variants in the *DC-SIGN *promoter in Cantonese population.

Therefore, we explored the relationship between *DC-SIGN *promoter polymorphisms and susceptibility to NPC by determining *DC-SIGN *promoter sequence variation in a case-control study in Cantonese.

## Methods

### Study subjects

All subjects were unrelated Cantonese population in Guangdong Province, China. Cases were recruited consecutively from December 2005 to October 2006 with pathologically confirmed diagnosis of NPC at the Sun Yat-Sen University Cancer Center (SYSUCC), Guangzhou, China. Total 500 NPC patients were collected and 444 were Cantonese origin living in Southern China. Population controls were cancer-free individuals, randomly selected from individuals who attend annual community-based physical examinations during the same time period. The selection criteria for control subjects included no individual history of cancer, all of them were Cantonese, and matched to NPC cases by age (± 5 years), sex and the time period for blood sample collection. Total 464 controls were involved. All study subjects had signed informed consent agreements before epidemiological data and blood samples were collected by trained SYSUCC staff interviewers.

For both cases and controls, venous blood specimens totalling 5-10 ml were collected from subjects and genomic DNA was then extracted from the lymphocytes using the QIAamp DNA Blood Midi Kit (Qiagen, German) following the manufacturer's protocol. These procedures were reviewed and approved by the Human Ethics Approval Committee of SYSUCC.

### Genotyping of *DC-SIGN *promoter variants

A region approximately 1,041 bp upstream of the ATG start codon that includes the promoter region was amplified using the following primers: 5- 'GCAGTCTTGGTTCCTTGGAG -3' for forward primer 1 and 5- 'ACTTGCAGTGCCTCCTCAGT -3' for reverse primer 1; 5'-TGCTGCTGTCCTCATTTTTG-3' for forward primer 2 and 5'-AGCATACAGAAACCCCGTTG-3' for reverse primer 2. Primer 1 delimits the promoter region between nt -602 and 28 [GenBank: NC_000019.9)] and amplifies a 630 bp fragment. Primer 2 delimits the promoter region between nt -404 and nt -1041 and amplifies a 638 bp fragment. Polymerase chain reaction (PCR) amplification was performed in a volume of 20 μL as follows: 15.85 μL ddH_2_O, 2.0 μL 10 × reaction buffer (with Mg^2+^), 0.5 μL 4 × dNTP (10 mmol L^-1^), 0.2 μL of each primer (20 μM), 1.25 U Taq DNA polymerase, and 1 μL genomic DNA (20 ng). Touchdown PCR was performed in the model 9700 GeneAmp PCR system (Applied Biosystems, Foster City, CA) with the following conditions: one initial denaturation of 95°C for 5 minutes, and then 5 cycles of 94°C for 30 seconds, 61°C for 30 seconds (-0.5°C every cycle), and 72°C for 45 seconds; then 32 cycles of 94°C for 30 seconds, 60°C for 30 seconds, and 72°C for 45 seconds, followed by one elongation step at 72°C for 10 minutes. The amplified products were analysed by 1.5% agarose gel electrophoresis followed by ethidium bromide staining. PCR amplification was performed using the same conditions for primer 1 and primer 2 as described above.

PCR products were recovered, and further purified. Sequencing reactions were performed using PCR primers and all nucleotide sequences were obtained using the 3730 automated sequencer (Applied Biosystems). Sequence alignment and SNP search were inspected using DNAStar analysis programs (DNAStar, Madison, WI, USA) using the nucleic acid sequences from Genebank at National Center for Biotechnology Information (NCBI) as the prototype sequence [GenBank: NC_000019.9].

### Statistical analysis

To test the different promoter polymorphisms of *DC-SIGN *gene for a possible distortion in genotypic and allelic frequencies between cases and controls, a chi-square test or Fisher's exact test was used to compare the genotypic and allelic distribution between cases and controls. In addition, to control confounding factors, unconditional logistic regression analysis was conducted to compare the genotype frequencies between cases and controls by adjusting for age, sex, the level of educational. The analyses were performed in SPSS software for Windows, version 13.0 (SPSS). For power calculation, the QUANTO program (Version 1.2) was used.

Haplotype frequencies were estimated using the accelerated expectation maximisation algorithm implemented in Haploview V 4.0 [36]. Haplotype frequencies occurring at <5% were excluded from the analysis. Association testing for the haplotypes was performed using the chi-square test. ORs were calculated with 95% CI. Significant associations were defined as *P *< 0.05 and all statistical tests were two-tailed.

## Results

This study included a total of 444 NPC patients and 464 control subjects. The characteristics of study subjects are summarised in Table 1. The mean age (± standard deviation) of the patients was 41.7 ± 9.6 y and 61.0% of the cases were male. For controls, the age was 41.3 ± 10.6 y and 60.1% of subjects were male. There was no statistically significant difference between cases and controls with respect to the frequency distributions of age and the sex distribution was unbiased (all *P*-values > 0.05). There was significant association between the level of education and NPC susceptibility. In current study, 21.7% of the case patients versus 8.0% of control subjects were illiterate or had an education of primary school (*P *< 0.001).

**Table 1:** Demographic characteristics and socio-economic status of the study population

Variables	Case (n = 444)	Control (n = 464)	*P*-value*
Gender (%)			
Male	271 (61.0)	279 (60.1)	0.78
Female	173 (39.0)	185 (39.9)	
Age(%), mean ± SD (y)	41.7 ± 9.6	41.3 ± 10.6	
≤30	49 (11.0)	61(13.1)	0.71
31-40	163 (36.7)	158 (34.1)	
41-50	162 (36.5)	163 (35.2)	
51-60	51 (11.5)	60 (12.9)	
61~	19 (4.3)	22 (4.8)	
Level of education			
Illiteracy or primary school	94 (21.7)	37 (8.0)	<0.001
High school	268 (61.8)	255 (55.1)	
University or above	72 (16.6)	171 (36.9)	

*P- values were calculated using the chi-square test.

Direct sequencing of the promoter region of *DC-SIGN *revealed the occurrence of six variants: -939 G/A, -871 A/G, -336 A/G, -190 A/G, -139 A/G and -116 G/T. Table 2 presents detailed information on these six variants. The minor allele frequencies (MAFs) of all SNPs evaluated in the *DC-SIGN *promoter were >0.05 except for -190 A/G (MAF = 0.003) and -116 G/T (MAF = 0.006), and all loci fit Hardy-Weinberg equilibrium expectations (*P*-values > 0.05).

**Table 2:** The minor allele frequencies and Hardy-Weinberg equilibrium tests of 6 variants in *DC-SIGN *gene

Polymorphism		NCBI rs number	**Position**^**†**^	**Genotype**^**‡**^	Gene region	**MAF **^**§**^	**PH-W**^**¶**^	% Geno -typed
							
Code	Name							
1	*DC-SIGN *-116	--	7718513	G/T	Promoter	0.006	1.00	100.0
2	*DC-SIGN *-139	rs2287886	7718536	A/G	Promoter	0.275	0.64	100.0
3	*DC-SIGN *-190	--	7718587	A/G	Promoter	0.003	1.00	100.0
4	*DC-SIGN *-336	rs4804803	7718733	A/G	Promoter	0.085	0.96	100.0
5	*DC-SIGN *-871	rs735239	7719268	A/G	5'flanking	0.154	0.58	100.0
6	*DC-SIGN *-939	rs735240	7719336	G/A	5'flanking	0.222	0.71	100.0

^†^The chromosome position listed here is taken from the NCBI database dbSNP build 130.

^‡ ^First allele is major allele, the second is minor allele.

^§ ^MAF denotes Minor Allele Frequency.

^¶^P_H-W _represents the *P *value of Hardy-Weinberg equilibrium tests.

Table 3 presents the genotypic and allelic frequencies of these six variants among Cantonese population. For *DC-SIGN *-139 A/G, subjects carrying mutant genotype -139 AG, which means a SNP -139 A/G in heterozygous alleles and -139 GG, which means a SNP -139 A/G in both G alleles, had 1.42-fold (95% CI = 1.07-1.86) and 1.99-fold (95% CI = 1.20-3.30) increased risks of developing NPC, respectively, when compared with those carrying wild genotype -139 AA. After adjusting for age, sex and the level of education, genotype -139 AG and -139 GG had 1.41-fold (95% CI = 1.05-1.88) and 2.10-fold (95% CI = 1.23-3.59) increased risks of developing NPC, respectively, when compared with those carrying wild genotype -139 AA (*P *_trend _= 0.005). Furthermore, subjects carrying the risk allele -139 G had 1.42-fold (95% CI = 1.15-1.74) increased risks of developing NPC when compared with those carrying allele -139 A (*P *= 0.001). In addition, subjects possessing mutant genotype -871 AG and GG had higher risks of developing NPC (OR = 1.43; 95% CI = 1.06-1.93 and OR = 2.34; 95% CI = 0.99-5.54, respectively) when compared with those carrying wild genotype -871 AA and those carrying the risk allele -871 G had 1.47-fold (95% CI = 1.14-1.90) increased risks of developing NPC when compared with those carrying allele -871 A (*P *= 0.003). After adjusting for age, sex and the level of education, there was no significant association between -871 GG genotype and NPC susceptibility (*P *= 0.14), which maybe mainly due to the small sample size. However, the association between -871 AG genotype and NPC susceptibility was still significant (*P *= 0.03). For *DC-SIGN *-939 G/A, subjects carrying mutant genotype -939 AA had a 2.56-fold increased risk of developing NPC (OR = 2.56; 95% CI = 1.36-4.83; *P *= 0.003) compared to those carrying wild-type genotype -939 GG and the association is still significant after adjusting for age, sex and the level of education (OR = 2.52; 95% CI = 1.29-4.93; *P *= 0.007). Furthermore, the association between the risk allele -939 A and NPC susceptibility was statistically significant (OR = 1.43; 95% CI = 1.15-1.79; *P *= 0.002). However, for the other three SNPs, there were no significant differences between cases and controls. For power calculation, the powers for all of the three significant SNPs, DC-SIGN-139, -871 and -939, were 0.92, 0.87 and 0.90, respectively.

**Table 3:** Association between *DC-SIGN *promoter variants and NPC

SNP	Genotype	Case	Control	UnadjustedOR (95% CI)*	Unadjusted *P*-value*	**Adjusted OR (95% CI) **^**#**^	**Adjusted *P-*value **^**#**^
*DC-SIGN *-116	GG	440	457	ref		ref	
	GT	4	7	0.59(0.17-2.04)	0.40	0.57 (0.16-2.01)	0.38
	TT	0	0	--	--	--	--
Allele	G	884	921	ref			
	T	4	7	0.60(0.17-2.04)	0.40	--	--
*DC-SIGN *-139	AA	212	268	ref		ref	
	AG	188	168	1.42 (1.07-1.86)	0.01	1.41 (1.05-1.88)	0.02
	GG	44	28	1.99 (1.20-3.30)	0.007	2.10 (1.23-3.59)	0.006
							*P *_trend _= 0.005
Allele	A	612	704	ref			
	G	276	224	1.42 (1.15-1.74)	0.001	--	--
*DC-SIGN *-190	AA	441	462	ref		ref	
	AG	3	2	1.57 (0.26-9.45)	0.68	1.57 (0.24-10.36)	0.64
	GG	0	0	--	--	--	--
Allele	A	885	926	ref			
	G	3	2	1.57 (0.26-9.42)	0.68	--	--
*DC-SIGN *-336	AA	365	396	ref		ref	
	AG	77	63	1.33 (0.92-1.91)	0.13	1.43 (0.98-2.09)	0.07
	GG	2	5	0.43 (0.08-2.25)	0.45	0.57 (0.11-3.07)	0.51
							*P *_trend _= 0.14
Allele	A	807	855	ref			
	G	81	73	1.18 (0.85-1.64)	0.34	--	--
*DC-SIGN *-871	AA	301	352	ref		ref	
	AG	127	104	1.43(1.06-1.93)	0.02	1.44 (1.05-1.98)	0.03
	GG	16	8	2.34 (0.99-5.54)	0.05	1.97 (0.80-4.88)	0.14
							*P *_trend _= 0.04
Allele	A	729	808	ref			
	G	159	120	1.47 (1.14-1.90)	0.003	--	--
*DC-SIGN *-939	GG	251	301	ref		ref	
	AG	161	148	1.31 (0.99-1.72)	0.06	1.28 (0.95-1.71)	0.11
	AA	32	15	2.56 (1.36-4.83)	0.003	2.52 (1.29-4.93)	0.007
							*P *_trend _= 0.01
Allele	G	663	750	ref			
	A	225	178	1.43 (1.15-1.79)	0.002	--	--

* Odds ratios and *P- *values were calculated using the chi-square test.

^# ^Odds ratios and P-Values were calculated by adjusting for age, sex, educational level.

**Figure 1:**
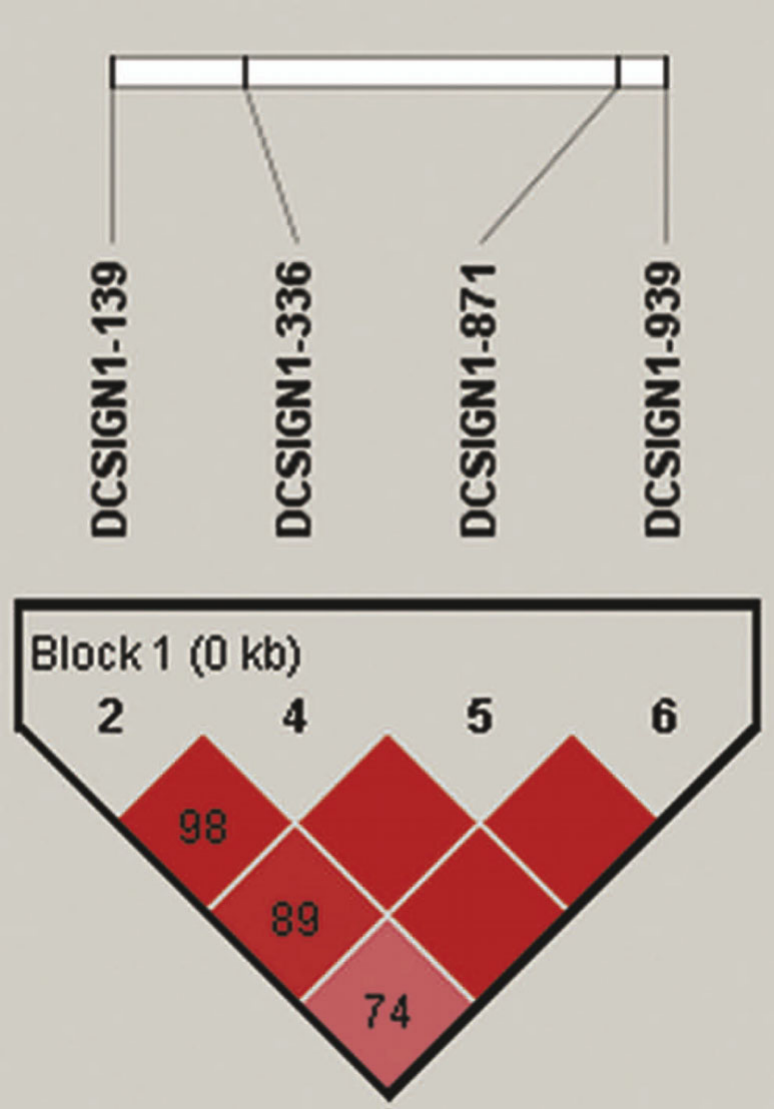
**LD block structure around DC-SIGN promoter region**. Haplotype block structure, as depicted by Haploview, is shown. The three-color scheme (white to red) represents the increasing strength of LD. Values for D' is shown, those boxes with D' = 1 are shaded in bright red and blank. Cells with D' < 1 are shades of pink or red with 100 × D' indicated.

Moreover, we performed the analyses at the haplotype level and found that there was significant linkage disequilibrium among *DC-SIGN *-139 A/G, -336 A/G, -871 A/G and -939 G/A, and a block was constructed by these four SNPs (Figure 1). Table 4 shows the results of haplotype analysis and reveals that h1 'AAAG', representing wild-type for all four common SNPs (-139 A, -336 A, -871 A and -939 G) and accounting for 68.4% of all haplotypes, was associated with protection against NPC (OR = 0.69; 95% CI = 0.57- 0.84; *P *= 2.0 × 10^-4^). Further, using 1000 permutation test runs, we also detected a significant association (*P *= 0.001) between this haplotype and NPC phenotype. In addition, h2 'GAGA', which accounts for 14.1% of all haplotypes, was associated with NPC risk (OR = 1.34; 95% CI = 1.03-1.75; *P *= 0.03). However, when using using 1000 permutation test runs, there was no significant association between h2 'GAGA' and NPC phenotype (*P *= 0.23).

**Table 4:** Association between *DC-SIGN *Haplotypes and NPC risk

**Hap**^**†**^	Block	**HaploFreq **^**‡**^	Case Ratios	Control Ratios	Chisq	OR (95% CI)	*P*-value*	**EMP **^**¶**^
h1	AAAG	0.684	0.643	0.723	13.6	0.69 (0.57-0.84)	2.0 × 10^-4^	0.001
h2	GAGA	0.141	0.159	0.124	4.70	1.34 (1.03-1.75)	0.03	0.23
h3	GGAG	0.084	0.091	0.078	1.06	1.19 (0.85-1.66)	0.30	0.92

^† ^The code of each haplotype.

^‡^The frequency of each haplotype.

*P- values were calculated using the chi-square test.

^¶^EMP = 1,000 permutation test P value.

## Discussion

This report on the distribution of genetic polymorphisms in the *DC-SIGN *promoter in the Cantonese population revealed that *DC-SIGN *-139 GG and -939 AA were significantly associated with increased risk of NPC and the risk allele *DC-SIGN *-871 G was significantly associated with NPC susceptibility (Table 3). Haplotype analysis revealed that h1 'AAAG', which contains all four wild-type SNPs (-139 A, -336 A, -871 A and -939 G), was associated with a significantly decreased risk of NPC and h2 'GAGA' was significantly associated with the NPC phenotype (Table 4). Moreover, we found two new variants in Cantonese population, -116 G/T and -190 A/G, although the MAFs for both were low (0.6% and 0.3%, respectively).

In current study, the MAFs of four common SNPs (-139 A/G, -336 A/G, -871 A/G and -939 G/A) in the promoter region of *DC-SIGN *in Cantonese population were similar to that in 45 unrelated Han Chinese in Beijing, China (HCB) http://www.ncbi.nlm.nih.gov/projects/SNP/snp. In addition, Kashima et al. [37] found that the allelic frequency of DC-SIGN -332 A was 10.7% by sequencing 28 Asians; however, this SNP was neither detected by Barreiro et al. in Asians [30] nor by Koizumi et al. in Japanese individuals [31]. Equally, this SNP is not detected in individuals of Cantonese in current study, which indicates that *DC-SIGN *-332 A is not widespread in most Asians. Two other SNPs in the promoter region, DC-SIGN -745 G/T and -201 G/T, were not present in current cohort, though they were observed exclusively in the Zimbabwean population [38]. The allelic distribution of DC-SIGN genes differs widely in populations from different ethnic groups, presumably the result of selective pressure exerted by prevalent pathogens in these geographically distinct regions. NPC is rare in most populations around the world but common in China and Southeast Asia and this could be reflected in SNP frequencies. *DC-SIGN*, a protein expressed on the surface of DCs, has recently received considerable attention in research on AIDS [33], dengue [25,35], tuberculosis [29,30] and Ulcerative Colitis [39]. Previous studies have indicated that *DC-SIGN *-336 G is associated with protection against dengue disease in Thailand population [25] and tuberculosis disease in sub-Saharan Africa individuals [29]. It may mainly due to the location of the *DC-SIGN *-336 SNP 214 bp upstream of the major transcription site, affecting a Sp1-like binding site and further modulating *DC-SIGN *transcriptional activity [25]. However, Barreiro and colleagues [30] found that -336 A and -871 G variants conferred protection against tuberculosis in Eurasian populations. Meriem Ben-Ali et al. [40] found no association between *DC-SIGN *promoter variation and susceptibility to tuberculosis in Tunisian patients. These contrasting results may be due to significant differences in the distribution of *DC-SIGN *alleles in different ethnic populations [38]. Different population genetic backgrounds as well as differences in linkage disequilibrium (LD) patterns can be at the basis of the conflicting results. In current study, there was no observable association between *DC-SIGN *-336 SNP and NPC susceptibility in the Cantonese population.

The mechanism of involvement of mutant *DC-SIGN *-139 and 939 in the pathogenesis of NPC remains unknown. Previous studies have already demonstrated a higher frequency of allele *DC-SIGN *-139 A in individuals not infected with HIV compared with infected patients [32]. In another study, allele -139 G was found to be associated with the rapid progression of AIDS in a population of Japanese haemophiliacs [31]. In current study, we found that the frequencies of *DC-SIGN *-139 GG and -939 AA were significantly higher in NPC patients compared with healthy controls. One potential mechanism for this effect may involve differential inducible expression of *DC-SIGN *on blood DCs as a result of these two polymorphisms, but this remains to be demonstrated. *DC-SIGN *-139 is located close to one of the binding sites of the transcription factor AP-1 in the promoter region of *DC-SIGN*, and we could speculate that the substitution of one nucleotide close to this site may change the level of expression of *DC-SIGN *and further contribute to the progression of NPC. As for *DC-SIGN *-939, it is yet to be determined whether this variant will affect the expression of *DC-SIGN*.

EBV is an important etiological agent of NPC and establishes persistent infections by employing multiple strategies to evade host immune responses. Consistent with the critical function of DCs in anti-viral immunity, myriad viruses are known to infect different subsets of DCs and to affect their differentiation, survival, and migration and/or T cell stimulatory capacity [41-44]. However, no studies have been performed so far to determine whether DC-SIGN is the EBV receptor. EBV has been observed to infect DC-SIGN positive cells such as immature DCs [45], monocytes [46-48] and some macrophages [49-51]. Furthermore, Li et al. [45]showed that EBV infection inhibited DC development from monocyte precursors, and further showed that immature DCs that become resistant to EBV-induced apoptosis still support virus entry [50]. Guerreiro-Cacais AO et al reported that EBV-infected macrophages could facilitate dissemination of EBV within the oral mucosal epithelium [50]. Recently, DC-SIGN could serve as putative receptor for secretory IgA (SIgA) on immature DCs by binding to high mannose glycoprotein on SIgA protein has been reported [52]. Sixbey JW et al have demonstrated that EBV-SIgA complex promoted EBV infection of epithelial cells through secretory component-mediated IgA transport [53]. We hypothesize that DC-SIGN expressed on immature DCs may recognize EBV-SIgA complex though binding to SIgA and thus promote EBV infection of immature DCs. This link DC-SIGN to EBV infection of immature DCs was need further confirmed and the study is on the way in our Lab.

We would like to point out that the sample size of the current study was not large enough and these results need to be validated in larger samples. Despite limitations, the current study represents the first comprehensive genetic association study examining the relationship between *DC-SIGN *promoter genetic variants and NPC risk in a case-control study and supplying genetic data of *DC-SIGN *promoter polymorphism in Cantonese population.

## Conclusions

Our study shows that the mutant genotypes -139 GG and -939 AA detected in the promoter region of the *DC-SIGN *gene were involved in NPC susceptibility, and further studies are necessary to demonstrate the role of *DC-SIGN *promoter polymorphisms in the function of *DC-SIGN *as well as their effect on EBV infection.

## Competing interests

The authors declare that they have no competing interests.

## Authors' contributions

Designed the study: WHJ and MSZ; collected samples and experiments: WHJ, QSF, LZC, WSL, YFX, JQD, WLL, YXZ; performed the data analysis: YFX; writing the manuscript: YFX, WHJ and MSZ.

All authors read and approved the final manuscript.

## Pre-publication history

The pre-publication history for this paper can be accessed here:

http://www.biomedcentral.com/1471-2350/11/161/prepub
